# Characterization and Application of EST-SSR Markers Developed From the Transcriptome of *Amentotaxus argotaenia* (Taxaceae), a Relict Vulnerable Conifer

**DOI:** 10.3389/fgene.2019.01014

**Published:** 2019-10-18

**Authors:** Xiaoxian Ruan, Zhen Wang, Ting Wang, Yingjuan Su

**Affiliations:** ^1^School of Life Sciences, Sun Yat-sen University, Guangzhou, China; ^2^College of Life Sciences, South China Agricultural University, Guangzhou, China; ^3^Research Institute of Sun Yat-sen University, Shenzhen, China

**Keywords:** *Amentotaxus argotaenia*, transcriptome, EST-SSR, genetic variation, population structure

## Abstract

*Amentotaxus argotaenia* (Taxaceae) is a vulnerable coniferous species with preference for shade and moist environment. Accurate estimation of genetic variation is crucial for its conservation, especially in the context of global warming. In this study, we acquired a transcriptome from *A. argotaenia* leaves using Illumina sequencing and *de novo* assembled 62,896 unigenes, of which 5510 EST-SSRs were detected. Twenty-two polymorphic EST-SSRs were successfully developed and further used to investigate genetic variation, linkage disequilibrium, and bottleneck signatures of *A. argotaenia*. The results showed that *A. argotaenia* had moderate genetic variation and high genetic differentiation, which may provide raw material to protect against climatic changes and accelerate local adaptation, respectively. No bottlenecks were found to occur in *A. argotaenia*. Our study not only showed that these EST markers are very effective in population genetic analysis but also lay a solid foundation for further investigating adaptive evolution and conservation strategies of *A. argotaenia*.

## Introduction

The genus *Amentotaxus* Pilger (Taxaceae) has six species, and five of them have been listed as endangered, vulnerable, or near threatened (Fu et al., 1999a; Farjon and Filer, 2013; Hilton-Taylor et al., 2013). Among all *Amentotaxus* species, *Amentotaxus argotaenia* (Hance) Pilger has the widest distribution but with small isolated populations occurring in southern and central China, northern Vietnam, and Laos (Farjon and Filer, 2013). Its preferred environments are limestone mountains, forests, ravines, and shady and damp stream banks, at altitudes of 300–1100 m (Fu et al., 1999a; Lin et al., 2007). The natural regeneration of the plant is infrequent due to slow growth rate and poorly dispersed seeds. Moreover, forest clearing and habitat loss have also been severely reducing its population size. *A. argotaenia* is listed as vulnerable in China and as near threatened in the International Union for Conservation of Nature Red List of Threatened Species (Hilton-Taylor et al., 2013). Since knowledge of population genetics is essential for the conservation and sustainable use of wild resources (Wayne and Morin, 2004; Hoban et al., 2013), we aim to examine the population genetic variation of *A. argotaenia* using novel molecular markers.

In comparison to genomic SSRs, expressed sequence tag (EST)-SSRs represent functional markers that may linked with functional genes inducing phenotypic effects (Ranade et al., 2014; Zhou et al., 2018a). They hence provide opportunities to examine functional diversity in relation to adaptive variation. Moreover, EST-SSRs are reported to be more reliable because they present lower frequencies of null alleles than do genomic SSRs (Yu and Li, 2008). With the advances in next-generation sequencing technology, EST-SSRs are becoming amenable to be identified by sequencing the transcriptomes (Zhou et al., 2018b). Next-generation sequencing is faster and more cost-effective than traditional approach, e.g., cDNA library construction method (Huang et al., 2015). However, currently, there is a shortage of EST-SSRs developed from *A. argotaenia*, although EST-SSRs have been identified for its congeneric species, *Amentotaxus formosana* (Li et al., 2016). Excavation and characterization of EST-SSRs for *A. argotaenia* may contribute to enhance our understanding of its population genetic diversity, structure, and the genetic basis of adaptive divergence. In addition, it will also provide resources to assess the association between transposable elements and SSR distribution as well as their roles in genome organization (La Rota et al., 2005; Wang et al., 2018a).

In this study, we constructed a leaf transcriptome of *A. argotaenia* using the Illumina sequencing platform. Based on the transcriptome sequencing data, we developed a set of EST-SSR markers and examined their polymorphisms. We then assessed the genetic variation of four populations of *A. argotaenia* using the novel EST-SSR markers. This work provides essential information for the conservation and management of *A. argotaenia* in the future.

## Materials and Methods

### Plant Materials and DNA Extraction

A total of 56 A*. argotaenia* individuals were sampled from four of its natural populations Jiuqushui (JQS; n = 15), Chuanping (CP; n = 13), Qiniangshan (QNS; n = 12), and Wugongshan (WGS; n = 16) located in China (**Supplementary Table 1**). Fresh leaves were collected and desiccated in sealed plastic bags with silica gel. Genomic DNA was isolated using the modified cetyltrimethylammonium bromide method (Su et al., 2005). DNA quality was evaluated using gel electrophoresis on 0.8% agarose gel.

### RNA Extraction, cDNA Library Construction, and Transcriptome Sequencing

Fresh young leaves of one *A. argotaenia* individual from population CP, which is planted in a greenhouse at Sun Yat-sen University, were used to extract total RNAs using the method described by Fu et al. (2004). RNA integrity was evaluated on agarose gels followed by quantification on an Agilent 2100 Bioanalyzer (Agilent Technologies, Santa Clara, California, USA). The mRNAs were isolated from the total RNAs by using a Dynabeads mRNA DIRECT Kit (Invitrogen Life Technologies, Carlsbad, California, USA) and randomly fragmented. The fragmented mRNAs were converted into double-stranded cDNA by using random primers and reverse transcriptase. After end-repairing and tailing A, the cDNA fragments were ligated to Illumina paired-end adapters. The cDNA library was sequenced on an Illumina Hiseq2500 platform (Illumina, San Diego, California, USA) with insertion size of 400–500 bp.

### Transcriptome Assembly, Functional Annotation, and Classification

We obtained a total of 25,257,542 paired-end reads from *A. argotaenia*. The reads were filtered by removing primer or adaptor sequences, and reads that contain unknown (“N”) or poor-quality bases (the mean quality per base < 15 with a 4-base wide sliding window) using the Trimmomatic software version 0.32 (Bolger et al., 2014). The resulting clean data were deposited in Sequence Read Archive of the National Center for Biotechnology Information (NCBI) (Bioproject no. PRJNA413732; Biosample no. SAMN07764634; https://www.ncbi.nlm.nih.gov/bioproject/PRJNA413732). The clean reads longer than 90 nt were *de novo* assembled into contigs and transcripts using the TRINITY software (https://github.com/trinityrnaseq/trinityrnaseq/releases) with default settings. The transcripts that cannot be prolonged at either end were defined as unigenes.

All unigenes were searched against Nt (NCBI nucleotide sequences), Nr (NCBI non-redundant database), and Swiss-Prot (a manually annotated and reviewed protein sequence database) through blast 2.2.30+ (ftp://ftp.ncbi.nlm.nih.gov/blast/executables/blast+/2.2.30/) with a cut-off *E*-value of 10^−5^. Protein domains of open reading frame within unigenes was identified by using HMMER hmmscan (hmmer-3.1b2-linux-intel-x86_64) and Pfam (the protein families database). Blast2GO version 3.0 was used to perform Gene Ontology (GO) annotations defined by molecular function, cellular component, and biological process ontologies (http://www.blast2go.com/b2ghome). We further used the Kyoto Encyclopedia of Genes and Genomes (KEGG) database to performed pathways annotation and euKaryotic Ortholog Groups (KOG) database to predict possible functions.

### Development of EST-SSRs

EST-SSRs were searched in the assembled unigenes using MIcroSAtellite (http://pgrc.ipk-gatersleben.de/misa/misa.html). The SSRs were assumed to contain mono-, di-, tri-, tetra-, penta-, and hexa-nucleotides with minimum repeat numbers of 10, 6, 5, 5, 5, and 5, respectively. Primer premier 5.0 (Clarke and Gorley, 2001) was used to design primers. Only those SSRs containing two to six repeat motifs were considered. Major parameters for primer design included the following: (1) primer length ranging from 16 to 27 bp, (2) GC content of 30%–70%, (3) melting temperature between 50 and 63°C, and (4) PCR product size ranging from 100 to 400 bp. PCR reactions were performed in 20 μl mixture containing 2 μl 10 × PCR buffer (Mg^2+^), 0.4 μl 10 mM dNTPs, 0.5 μl 10 mM each of primers, 1.25 U *Taq* polymerase, and 20 ng DNA template. The PCR protocol was as follows: 94°C for 5 min, followed by 30 cycles of 40 s at 94°C, 40 s at optimal annealing temperature and 30 s at 72°C, and a final elongation at 72°C for 10 min. Ampliﬁed products were screened on a 6.0% denaturing polyacrylamide gel, and fragment size was determined with 50-bp marker.

### Evaluation of Polymorphic EST-SSRs and Population Genetic Analysis

GenAlex software (Peakall and Smouse, 2006) was used to calculate the EST-SSR genetic parameters, including the number of observed alleles (*Na*), the effective number of alleles (*Ne*), observed heterozygosities (*Ho*), expected heterozygosities (*He*), and probability of the deviation from the Hardy–Weinberg equilibrium. The polymorphism information content (PIC) value and null alleles were evaluated using CERVUS 3.0 (Kalinowski et al., 2007) and Micro-Checker (Van Oosterhout et al., 2004), respectively. Linkage disequilibrium across loci was determined using TASSEL version 3.0 (Bradbury et al., 2007) with squared correlation coefficient (*r* *^2^* > 0.3) and the threshold of *p* values (< 0.001) based on Fisher’s exact test.

Arlequin version 3.5 (Excoffier and Lischer, 2010) was used to perform a Mantel test with 10,000 permutations to examine the pattern of isolation by distance. Using the same software, analysis of molecular variance was conducted to determine the amount of genetic variation at different levels.

Using EST-SSR data, we applied a Bayesian model-based clustering algorithm implemented in STRUCTURE 2.2 to infer population structure. We used the admixture model, setting the parameters as follows: burn-in periods = 10,000, MCMC replicates = 10,000, *K* = 1 to 8, and iterations = 10. The optimum number of clusters (*K*) was determined by calculating Δ*K* (Evanno et al., 2005).

We conducted the Wilcoxon’s sign-rank test and the mode-shift test to detect signatures of genetic bottleneck by running BOTTLENECK version 1.2.02 (Piry et al., 1999). The two-phase mutation model was selected because it is more suitable for microsatellite data than the other two models (Piry et al., 1999; Zhang and Zhou, 2013). We performed 1000 simulations under the two-phase mutation model with 70% single-step mutations and 30% multi-step mutations.

## Results

### Transcriptome Sequencing and *De Novo* Assembly

Approximately 23.5 million clean reads were obtained from the transcriptome of *A. argotaenia* with the length of 90–125 bp and GC content of 47%. The percentages of Q20 (base sequencing error probability < 1%) and Q30 (base sequencing error probability < 0.1%) bases were 100% and 97%, respectively. These clean reads were assembled into 80674 transcripts by using Trinity with an average length of 756 bp and an N50 of 1018 bp. The total length of transcripts reached 60,999,479 bp. After further assembly, a total of 62,896 unigenes were identified with an average length of 721 bp, a minimal length of 301 bp, and an N50 value of 947 bp. The sum of the length of the unigenes was 45,357,136 bp (**Table 1**). The length of 37.473% (23569) of the unigenes ranged from 301 to 400 bp, 61.956% (38,968) varied from 401 to 3000 bp, while 0.571% (359) was longer than 3000 bp (**Supplementary Figure 1**).

**Table 1:** Sequencing, assembly, and annotation results of *Amentotaxus argotaenia* transcriptome.

Total raw reads	25,257,542
Total clean reads	23,553,846
% Q20	100
% Q30	97
% GC	47
Number of transcripts	80,674
Average length of transcripts (bp)	756
N50 of transcripts (bp)	1018
Number of unigenes	62,896
Minimum length of unigenes (bp)	301
Average length of unigenes (bp)	721
N50 of unigenes (bp)	947
Number of unigenes annotated at least one databases	36,671
Number of unigenes with Nt annotations	13,140
Number of unigenes with Nr annotations	32,183
Number of unigenes with KOG annotations	32,953
Number of unigenes assigned to GO terms	31,283
Number of unigenes with Swiss-Prot annotations	26,309
Number of unigenes with Pfam annotations	21,595
Number of unigenes with KEGG pathways	15,072
Number of unigenes annotated to seven public databases	490

Nt, NCBI nucleotide sequences; Nr, NCBI non-redundant database; KOG, euKaryotic Ortholog Groups; GO, Gene Ontology; KEGG, Kyoto Encyclopedia of Genes and Genomes.

### Functional Annotation and Categorization

We conducted the annotation of 62,896 unigenes in the seven public databases (Nr, Nt, KOG, Swiss-Prot, Pfam, KEGG, and GO), of which 36,671 were successfully annotated (**Table 1**). Of them, 13,140 (20.89%) were annotated in Nt, 32,183 (51.17%) in Nr, 26,309 (41.83%) in Swiss-Prot, 21,595 (34.33%) in Pfam, 31,283 (49.74%) in GO, 32,953 (52.39%) in KOG, and 15,072 (23.96%) in KEGG. A total of 490 unigene were identified in all seven databases (**Table 1**).

**Figure 1:**
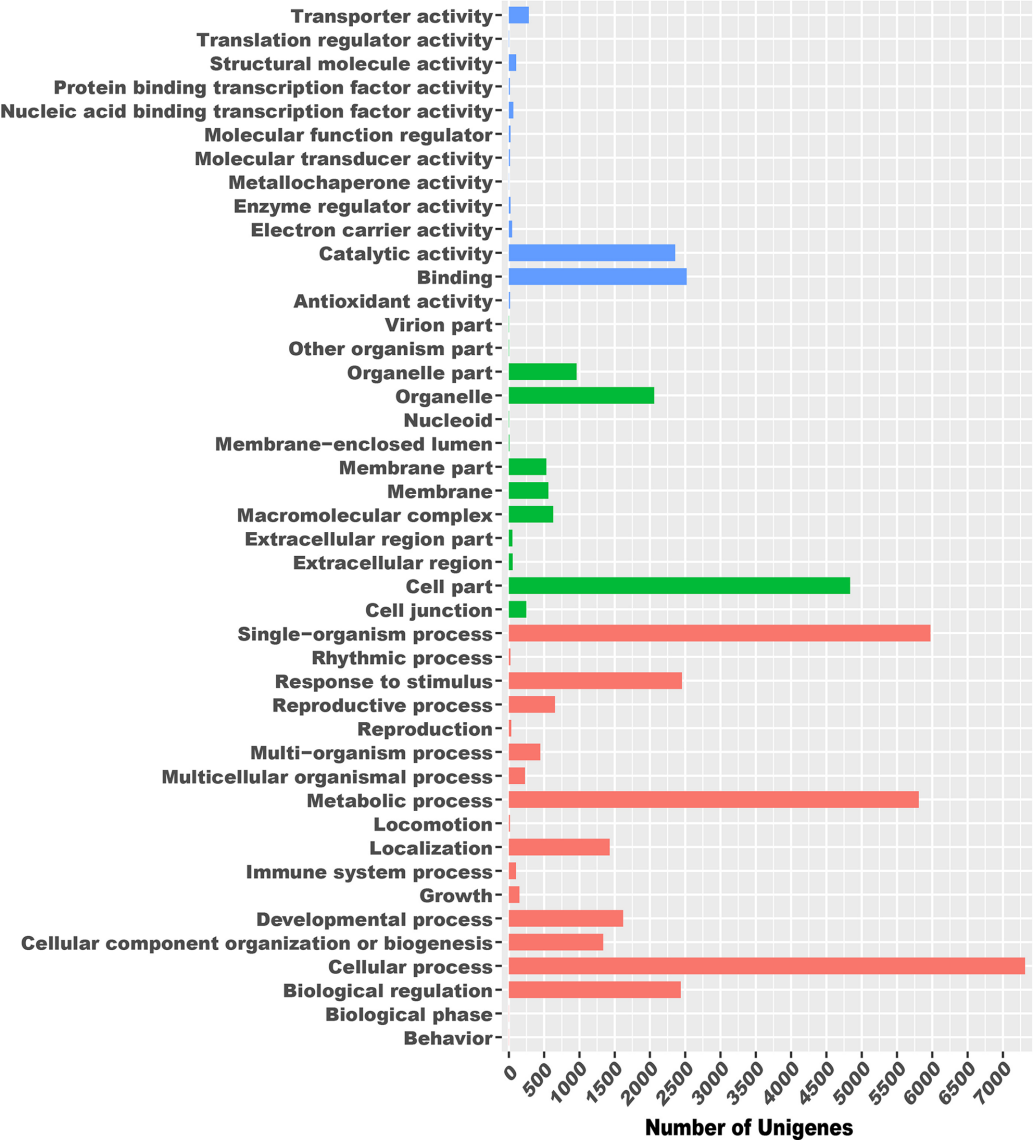
Functional classification of Gene Ontology for *Amentotaxus argotaenia* unigenes.

First, 31,283 unigenes were classified into three main GO categories: biological process, cellular component, and molecular function, including 44 functional groups. In the biological process category, there were 7313 unigenes assigned to “cellular process,” 5973 to “single-organism process,” 5808 to “metabolic process,” and 1 to “biological regulation.” In the cellular component category, “cell part” and “organelle” component-related functions were predominant, with 4835 unigenes assigned to the former and 2060 to the latter. In the molecular function category, “binding” and “catalytic activity” were the most enriched, comprising 2520 and 2358 unigenes, respectively (**Figure 1**).

Second, 32,953 unigenes were assigned to 25 KOG classifications. Among them, the largest group was “general function prediction only” (4286), followed by “function unknown” (3218), “signal transduction mechanisms” (2521), and “posttranslational modification, protein turnover, chaperones” (2442) (**Supplementary Figure 2**).

Third, a total of 15,072 unigenes were mapped to 38 KEGG pathways corresponding to six categories (**Supplementary Figure 3**). Most of them involved in “translation” (3007 unigenes; 19.95%), “carbohydrate metabolism” (2815; 18.68%), and “folding” (2425; 16.09%) pathways. The 38 KEGG pathways were related to such categories as metabolism, cellular processes, genetic information processing, environmental information processing, and others.

### Characterization of EST-SSRs

We identified 5510 EST-SSRs from 4830 SSR-containing unigene sequences, of which 362 were compound SSRs. With the exception of mononucleotide repeats (64.50%), trinucleotide repeats were the most common type with a frequency of 22.25%, followed by di- (9.18%), tetra- (1.82%), penta- (1.00%), and hexanucleotide (1.25%) repeats (**Table 2**). EST-SSRs with 10 tandem repeats (1719, 31.19%) were the most common, followed by >11 tandem repeats (1134; 20.58%) and 9 tandem repeats (42; 0.76%) (**Table 2**). Motif A/T (98.11%) was dominant in mononucleotide repeats, whereas AT/TA (50.79%) was the most abundant in dinucleotide repeats, followed by AG/CT (35.18%) and AC/GT (14.03%). Trinucleotide motifs AAG/CTT, AGG/CCT, AGC/CTG, AAT/ATT, and ACT/AGT had frequencies of 21.21%, 17.94%, 15.42%, 14.68%, and 0.33%, respectively.

**Table 2:** The distribution of repeat unit number and motif length of EST-SSRs.

SSR motif length					Repeat unit number					
	5	6	7	8	9	10	11	> 11	Total	Percentage (%)
Mono						1682	787	1085	3554	64.50
Di		255	89	60	32	20	16	34	506	9.18
Tri	747	266	112	61	8	15	5	12	1226	22.25
Tetra	65	27	1	4	1	2			100	1.82
Penta	42	3	3	3			2	2	55	1.00
Hexa	34	18	5	8	1		2	1	69	1.25
Total	888	569	210	136	42	1719	812	1134	5510	
Percentage (%)	16.12	10.33	3.81	2.47	0.76	31.19	14.74	20.58		

Mono, mono-nucleotide; Di, di-nucleotide; Tri, tri-nucleotide; Tetra, tetra-nucleotide; Penta, penta-nucleotide; Hexa, hexa-nucleotide.

We also examined the linkage of the 22 EST-SSR loci. Of 231 combinations, four pairs of loci (*SHS-1306* vs *SHS-26840*, *SHS-1306* vs *SHS-38297*, *SHS-28717* vs *SHS-32587*, and *SHS-34629* vs *SHS-38297*) were found to be linked with *r* *^2^* > 0.3 and *p* < 0.001.

### Polymorphic EST-SSRs Identification and Estimation of Genetic Diversity

We randomly selected 60 EST-SSR primers to evaluate their application and the polymorphism across 12 A. *argotaenia* individuals from four populations. Twenty-two of the microsatellite loci exhibited allelic polymorphism, whereas 16 were identified as monomorphic (**Table 3**). We then used the 22 polymorphic EST-SSR markers to perform population genetic analysis (**Supplementary Figure 5**).

**Table 3:** Characterization of 38 EST-SSR primer pairs for *A. argotaenia*.

Locus	Primer sequences (5′–3′)	Repeat motif	Allele size(bp)	*T*_a_(°C)	GenBank accession no.	Putative function [Organism]	*E*-value
**SHS-1306**	F: ACCTCGGGTCCTGTTGAAR: GGTTGTGGCGAATGCTG	(TAC)_6_(TAT)_5_	249	54	MG209531	Unknown [*Picea sitchensis*]	3e-53
SHS-1845	F: TCTGAGATAAGGTGCTTGGGTGR: ATTTGAGGGCTACAGCGGTT	(GTC)_7_	126	55	MG209532	Unknown [*P. sitchensis*]	3e-48
**SHS-2019**	F: AGAGACCACCAACGACGAACR: CAGCGGCAGCATACCATT	(TCC)_7_	298	55	MG209533	hypothetical protein AMTR_s00032p00067030 [*Amborella trichopoda*]	3e-25
SHS-2589	F: CTAACCCTATCCCTAACCTCTTTCR: GTTTCATTCCAGGCACTCTCA	(GAA)_5_	159	57	MG209534	Unknown [*P. sitchensis*]	2e-121
SHS-5811	F: TAGATTTAGTTCCCAGCGGTGR: GATTGATTTCGGCTCGTGTAT	(AG)_8_	279	53	MG209535	Not found	—
**SHS-6181**	F: TTCTACTTCTGCTGCTGGTGTR: GCATTGGTCTTCCTCCTTTAC	(TTC)_7_	214	54	MG209536	hypothetical protein AMTR_s00003p00222410 [*A. trichopoda*]	0
**SHS-17706**	F: CTCTTTGGGAGAAGTATTAGCR: TGGTCACTCGTGGACATTA	(AAGA)_5_	134	53	MG209537	PREDICTED: uncharacterized protein LOC103491482 isoform X2 [*Cucumis melo*]	5e-45
SHS-18170	F: GAGAGCCCACGGTCCTGTR: AGTCCCATCATCCACCTATCA	(GAA)_6_	300	53	MG209538	PREDICTED: zinc finger protein 43-like [*Pyrus x bretschneideri*]	1e-10
**SHS-18213**	F: AAAGTCGGGTGATTACAGAGCR: TCCTTCGTGGAATGTTTATGA	(GA)_7_	397	54	MG209539	Not found	—
**SHS-18563**	F: ACCTCCTACACCCCCTTCTR: AACTCCACCATACGCATCTTA	(GAA)_6_	236	54	MG209540	Unknown [*P. sitchensis*]	6e-97
**SHS-19735**	F: CCCAAAGAAAGGGCAAGAR: CGGCGGATGGTAATGTG	(CGC)_7_	278	53	MG209541	Unknown [*P. sitchensis*]	3e-64
SHS-20137	F: CTGTCAGGCATTTCTGGGTCTR: CGATTTTCATTTTGTTTGGTCTG	(CTT)_7_	257	58	MG209542	Not found	—
**SHS-20198**	F: CATTCTCACACCCTTGTATTGCTR: CATCTTCACCATTTCTCTGTAGTCTT	(TA)_8_	259	58	MG209543	transcription factor AP2 [*Taxus cuspidata*]	8e-117
SHS-21264	F: CTCGTCCAAGAAGAACCATACR: CATCATAAACCACTTAGCAAATAC	(GAG)_6_	400	56	MG209544	PREDICTED: uncharacterized protein LOC104240103 [*Nicotiana sylvestris*]	7e-41
SHS-21490	F: GAGGAAGAGGGTTTTGGTCATR: AGTAGGCGTCTTTGGCGTT	(TAA)_6_	190	58	MG209545	hypothetical protein PRUPE_ppa010075mg [*Prunus persica*]	1e-59
SHS-22515	F: CACATCCTCCGCCGACTR: TTGCTGTTTTACCGAGAAGAAG	(TAC)_6_(TAT)_5_	266	57	MG209546	Unknown [*P. sitchensis*]	2e-53
SHS-23191	F: ACCCAGTTGTGGTAGGAGCATR: AAAGTGTGAAACATCCCAAAGC	(GAG)_6_	161	57	MG209547	Not found	—
SHS-23195	F: TGACAACGAGAACGAAGAACATAACR: GTCTGTAAGCCAACGCTGAGG	(AGA)_6_	115	57	MG209548	hypothetical protein SELMODRAFT_451322 [*Selaginella moellendorffii*]	2e-41
**SHS-24187**	F: CCTAATGGTGAATAACTTGTGCTCR: GCGAGTTTCTTGCTAAATGCTT	(TCTT)_5_	330	58	MG209549	Not found	—
SHS-24301	F: TACCTGACTGGACTGCTGAATR: ATGTTAGAGGAATACGATAGGCT	(CCG)_6_	377	57	MG209550	Unknown [*P. sitchensis*]	1e-32
**SHS-26622**	F: AGATACTCTTGTTTCAGGAGCATTR: CAACCCAGGACATCACCATAG	(AAG)_7_	228	57	MG209551	Not found	—
**SHS-26840**	F: GGGCGGAGGAGAATGGTCR: TGGGCTGCTGAAATAGGAAAC	(GGA)_6_	250	57	MG209552	PREDICTED: uncharacterized protein LOC104602979, partial [*Nelumbo nucifera*]	1e-65
**SHS-28207**	F: CAATCGGATAAGGTGTTTCTR: CGAATAGTGGTAATCAAATAGG	(GA)_16_	379	52	MG209553	Unknown [*P. sitchensis*]	0
SHS-28326	F: GTATGGAAGGGAGGCGAAATR: GCCGCTGTGGTTGTGAAG	(ATA)_6_	143	57	MG209554	Unknown [*P. sitchensis*]	4e-29
**SHS-28474**	F: AATAAGAATAGGAGGGGTGAAGACR: GAGACAGAGGATTTGTAACGGAG	(CGG)_6_	218	57	MG209555	PREDICTED: mediator of RNA polymerase II transcription subunit 30-like isoform X1 [*N. nucifera*]	1e-16
**SHS-28717**	F: CGTATCCCTGTTGATTCATTTTCR: GGTTGTATCATTCAGTCCCATTG	(GAAA)_6_	240	57	MG209556	Not found	—
SHS-30422	F: TTCCTTCTACTCCCTCTTCTATGTCR: AACTGCTTACCTAAATGGTGCTG	(CTT)_6_	163	57	MG209557	PREDICTED: probable cellulose synthase A catalytic subunit 5 [*Phoenix dactylifera*]	0
**SHS-31463**	F: ATGGATGGCAGGATTGGATR: AACAAATAAGGAAGAAGGTGGTAGT	(TTG)_6_	182	57	MG209558	Not found	—
SHS-31820	F: TTTGGTTCCATACCTGCTCCTR: TTCGTGGTCACTCTTTTCCCT	(GAT)_6_	149	57	MG209559	Unknown [*P. sitchensis*]	5e-172
**SHS-31908**	F: CCAGACTTGCCACATCAGCR: AACCCACAACCCACCAGAG	(ATG)_5_(AGG)_7_	395	57	MG209560	Unknown [*P. sitchensis*]	4e-11
**SHS-32587**	F: AAATGAGGAATAAGTAGGTGAAGTTR: GCACATTAGGGTTCCTGATTAC	(AGA)_6_	254	57	MG209561	Not found	—
**SHS-32686**	F: CAACCCGTCCCTTGCTTTAGR: CCTCTGCGTCCTTGTTGTTATC	(GGCAG)_5_	302	57	MG209562	Unknown [*P. sitchensis*]	6e-176
**SHS-32939**	F: TGGAAAAAACCACAGACGACTCR: GCCCTCAAACACAAAAGCAG	(ATA)_7_	143	56	MG209563	Unknown [*P. sitchensis*]	1e-94
**SHS-34629**	F: CTGGACAAAGAGAGCAACGGTR: AATGGCGACACAAGTGAGAAGT	(CTC)_7_	222	56	MG209564	Hypothetical protein AMTR_s00032p00067030 [*A. trichopoda*]	2e-19
SHS-35222	F: TGCTGCCTAAACACAATGTCTCTR: CACAAGTCTTCCTTTTCCCTAATG	(TG)_9_	121	56	MG209565	Not found	—
**SHS-35453**	F: GTTGAGCATTGATTTAGATGTTCGR: TTTCCCCTCCTCTTTCTTTGAC	(TGA)_8_	189	55	MG209566	PREDICTED: uncharacterized protein LOC104596414 isoform X2 [*N. nucifera*]	2e-68
**SHS-38297**	F: TTACCAACGCCAAATGCTGR: ACCCTACTCCCACTCCCTTCT	(GAGATG)_7_	154	55	MG209567	hypothetical protein AMTR_s00099p00142540 [*A. trichopoda*]	1e-24
SHS-39519	F: TTGTGCCTCTTCAAGGAGTAGTR: GAGAATCTTCCCTGTCGGTC	(CTC)_6_	261	55	MG209568	ACC synthase-like [*Picea glauca*]	0

T_a_, annealing temperature; bold font, polymorphic loci.

The number of observed alleles and the effective number of alleles varied from 1 to 7 and from 1 to 4.694 per locus, respectively. The observed heterozygosity ranged from 0 to 1.000 (average = 0.250), while the expected heterozygosity ranged from 0.000 to 0.787 (average = 0.390). The mean value of PIC was 0.455, with the minimum of 0.084 and the maximum of 0.707. Fourteen loci were identified as null allele. Six, 9, 14, and 16 EST-SSRs showed significant deviations from the Hardy–Weinberg equilibrium in populations JQS, CP, QNS, and WGS, respectively (**Table 4**).

**Table 4:** Genetic diversity statistics for four *A. argotaenia* populations based on 22 polymorphic EST-SSR primers.

	JQS (*N* = 15)				CP (*N* = 13)				QNS (*N* = 12)				WGS (*N* = 16)				
Locus	*N*_a_	*N*_e_	*H*_o_	*H*_e_	*N*_a_	*N*_e_	*H*_o_	*H*_e_	*N*_a_	*N*_e_	*H*_o_	*H*_e_	*N*_a_	*N*_e_	*H*_o_	*H*_e_	PIC
SHS-1306	2	1.923	0.000*^‡^	0.480	2	1.166	0.000*	0.142	2	1.180	0.000*	0.153	2	1.600	0.000*^‡^	0.375	0.395
SHS-2019	2	1.301	0.267	0.231	3	1.807	0.462	0.447	2	1.180	0.000*	0.153	2	1.133	0.000*	0.117	0.429
SHS-6181	1	1.000	0.000	0.000	1	1.000	0.000	0.000	2	1.087	0.083	0.080	3	2.327	0.000*^‡^	0.570	0.225
SHS-17706	3	1.822	0.600	0.451	4	1.931	0.231^‡^	0.482	3	2.880	0.500*	0.653	3	2.667	0.500*	0.625	0.605
SHS-18213	4	1.531	0.000*^‡^	0.347	2	1.742	0.000*^‡^	0.426	4	1.714	0.000*^‡^	0.417	3	1.910	0.000*^‡^	0.477	0.402
SHS-18563	1	1.000	0.000	0.000	1	1.000	0.000	0.000	2	1.800	0.000*^‡^	0.444	3	2.844	0.000*^‡^	0.648	0.371
SHS-19735	4	2.217	0.600	0.549	2	1.352	0.000*^‡^	0.260	2	1.385	0.000*^‡^	0.278	3	1.684	0.250*	0.406	0.393
SHS-20198	2	1.923	0.000*^‡^	0.480	3	1.610	0.000*^‡^	0.379	4	3.236	0.250*^‡^	0.691	3	2.032	0.000*^‡^	0.508	0.560
SHS-24187	4	2.284	0.467	0.562	3	2.048	0.692	0.512	3	2.667	0.667	0.625	4	2.090	0.375*	0.521	0.528
SHS-26622	6	4.091	0.800	0.756	7	4.694	1.000	0.787	4	2.743	1.000	0.635	4	2.498	1.000	0.600	0.707
SHS-26840	4	2.761	0.867	0.638	4	2.467	0.462	0.595	3	2.268	0.583	0.559	2	2.000	1.000*	0.500	0.514
SHS-28207	4	2.663	1.000	0.624	5	3.282	1.000	0.695	5	3.840	0.917*	0.740	3	1.684	0.500	0.406	0.701
SHS-28474	4	1.772	0.000*^‡^	0.436	3	1.610	0.000*^‡^	0.379	3	2.323	0.000*^‡^	0.569	4	1.707	0.000*^‡^	0.414	0.456
SHS-28717	2	1.471	0.000*^‡^	0.320	2	1.742	0.000*^‡^	0.426	3	2.323	0.000*^‡^	0.569	2	1.280	0.000*^‡^	0.219	0.581
SHS-31463	1	1.000	0.000	0.000	2	1.257	0.231	0.204	1	1.000	0.000	0.000	2	1.133	0.125	0.117	0.084
SHS-31908	1	1.000	0.000	0.000	3	1.857	0.000*^‡^	0.462	2	1.800	0.000*^‡^	0.444	3	1.293	0.000*^‡^	0.227	0.297
SHS-32587	2	1.471	0.000*^‡^	0.320	3	1.610	0.000*^‡^	0.379	4	2.595	0.083*^‡^	0.615	3	2.612	0.000*^‡^	0.617	0.534
SHS-32686	2	1.800	0.267	0.444	2	1.352	0.308	0.260	2	1.180	0.000*	0.153	2	1.969	0.125^‡^	0.492	0.399
SHS-32939	3	1.495	0.400	0.331	2	1.451	0.385	0.311	2	1.180	0.167	0.153	2	1.992	0.563	0.498	0.393
SHS-34629	2	1.301	0.267	0.231	2	1.649	0.538	0.393	2	1.385	0.000*^‡^	0.278	2	1.600	0.000*^‡^	0.375	0.473
SHS-35453	2	1.800	0.667	0.444	2	1.988	0.000*^‡^	0.497	2	1.882	0.583	0.469	3	2.667	0.500*	0.625	0.579
SHS-38297	2	1.301	0.267	0.231	2	1.451	0.385	0.311	2	1.087	0.083	0.080	1	1.000	0.000	0.000	0.384

N, number of individuals of each populations; N_a_, number of alleles per locus; N_e_, number of effective alleles per locus; H_o_, observed heterozygosity; H_e_, expected heterozygosity; PIC, polymorphism information content; *significant deviation from the Hardy–Weinberg equilibrium (p < 0.001); ^‡^significant possibility of the presence of null alleles detected by MICRO-CHECKER.

### Population Genetic Structure and Differentiation

Genetic differentiation (*F*st) based on EST-SSRs was 0.28198. Analysis of molecular variance revealed that 71.80% of the genetic variation occurred within populations, while 10.37% and 17.83% were attributed to among populations within groups and among groups, respectively (**Supplementary Table 2**). In addition, the result of Mantel test showed that there was no significant correlation between genetic and geographical distances (*p* = 0.3306).

**Figure 2:**
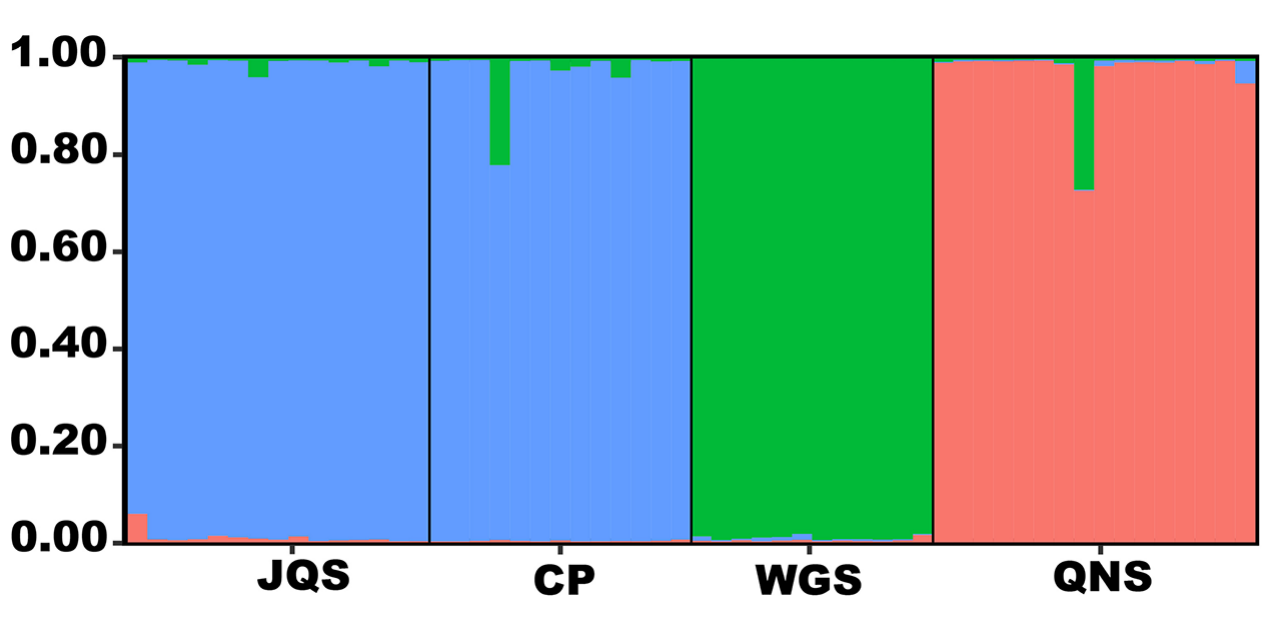
Population genetic structure analysis of *A. argotaenia* based on EST-SSRs. JQS, Population Jiuqushui; CP, Population Chuanping; WGS, Population Wugongshan; QNS, Population Qiniangshan.

Δ*K* demonstrated that the uppermost *K* equaled 3 (**Supplementary Figure 4**). *Amentataxus argotaenia* populations were assigned to three groups. Group I contained populations JQS and CP, group II contained population WGS, and group III contained population QNS (**Figure 2**).

### Bottleneck Signature

At species level, both Wilcoxon’s sign-rank test and mode-shift analysis indicated that *A. argotaenia* had not experienced a recent bottleneck (**Supplementary Table 3**).

## Discussion

### Characterization of Transcriptome

In this study, we sequenced, assembled, and annotated the transcriptome of *A. argotaenia* using the next-generation sequencing approach. A total of 62,896 unigenes were *de novo* assembled with the unigene mean and N50 length of 721 and 947 bp, respectively (**Table 1**). More than half of the unigenes can be successfully annotated through seven databases (Nr, Nt, KOG, Swiss-Prot, Pfam, KO, and GO), of which 490 were simultaneously identified (**Table 1**). Most of the annotated unigenes were unique in *A. argotaenia* compared to its closely related conifer *Torreya grandis* (Zeng et al., 2018). Moreover, the annotated results of NR database indicated that *A. argotaenia* exhibited only 27.5% unigene identity to another conifer *Picea sitchensis*. These unique unigenes may represent the species-specific genetic signature of *A. argotaenia* potentially underlying its speciation process or evolution (Frech and Chen, 2011). Similar results have been found in the case of *Picea abies* (Nystedt et al., 2013). In addition, 31,283, 32,953, and 15,072 unigenes of *A. argotaenia* were assigned into 44 functional groups in GO, 25 classifications in KOG, and 38 pathways in KEGG, respectively. These results indicate that the identified *A. argotaenia* unigenes have wide-ranging functions and will be valuable for analyzing the functional diversity of *A. argotaenia*.

### Frequency and Distribution of EST-SSRs

The mean length of unigenes (721 bp) of *A. argotaenia* was considerably longer than that of other conifers, including *Pinus pinaster* (495 bp) (Canales et al., 2014), *Platycladus orientalis* (686 bp) (Hu et al., 2016), and *P. abies* (472 bp) (Chen et al., 2012). Zalapa et al. (2012) pointed out that longer sequences will increase the probability to properly design EST-SSR primers. In accordance with this, we developed 5510 EST-SSRs from the transcriptome of *A. argotaenia*, which was significantly greater than those of *A. formosana* (4955) (Li et al., 2016) and *Pinus densiflora* (1953) (Liu et al., 2015). The most common motif for dinucleotides in *A. argotaenia* was found to be AT/TA. The same result was obtained in *Picea* spp. (Rungis et al., 2004), *Pinus dabeshanensis* (Xiang et al., 2015), and *Pseudolarix amabilis* (Geng et al., 2015). Ranade et al. (2014) emphasized that AT/TA was often ranked as the most abundant dimer motif in gymnosperms (especially in the 3′ untranslated regions). A factor related to this phenomenon may be increased A + T contents. In addition, the most common trinucleotide motif in *A. argotaenia* was AAG/CTT, which is similar to that in *Cryptomeria japonica* (Ueno et al., 2012), *Pinus taeda* (Wegrzyn et al., 2014), and *Pinus halepensis* (Pinosio et al., 2014), but unlike in *P. dabeshanensis* (AGC) (Xiang et al., 2015). It has been noted that AAG/CTT is the target for methylation in plants (Law and Jacobsen, 2010).

### Validation and Polymorphism of EST-SSRs and Population Genetic Variation

Thirty-eight of the 60 EST-SSR primers designed for *A. argotaenia* enabled to amplify expected products with a success rate of 63.33%, which is relatively high in comparison to previous studies (Dantas et al., 2015; Ueno et al., 2015). Then 22 polymorphic EST-SSRs were used to investigate standard genetic diversity and population genetic structure of four *A. argotaenia* populations. Only four pairs of them were tightly linked among each other. Moderate genetic variation was observed based on the EST-SSRs (PIC = 0.455) according to the evaluation criteria of polymorphism (moderate: 0.25 < PIC < 0.5) (Botstein et al., 1980). This judgment was also lent support by such genetic parameters as the average of observed alleles, observed heterozygosity, expected heterozygosity, and the percentage of polymorphic band. In contrast, previous researches detected low levels of genetic diversity in *A. argotaenia* by using ISSRs and genomic SSRs (Ge et al., 2005; Ge et al., 2015). These inconsistencies highlighted the importance to investigate genetic variation of *A. argotaenia* using multiple markers.

Accurate estimate of genetic diversity is very useful for conservation and management of genetic resources (Cardoso et al., 1998; Wang et al., 2018b). Compared to other conifers, we observed a moderate EST-SSR variation in *A. argotaenia* (**Supplementary Table 4**). Similar levels of the EST-SSR variation were found in its closely related species *A. formosana* as well (Li et al., 2016). The moderate level of functional diversity, together with the finding that *A. argotaenia* did not experience a recent bottleneck, implies that the species still has essential evolutionary potential to adapt to the changing environment (Frankham, 2010).

We detected a marked genetic differentiation among *A. argotaenia* populations in comparison to other conifers (**Supplementary Table 4**). Similar findings were obtained by using chloroplast intergenic spacer, mitochondrial intron, and genomic microsatellite data (Ge et al., 2015). The dispersal distance of pollen and seed in conifers is generally less than 2 and 20 km, respectively (Fu et al., 1999b; Cain et al., 2000; Sima, 2004; Zhang et al., 2005; Lu, 2006). As for *A. argotaenia*, its pollen and seed exchanges may be further hindered because of preferring to grow under forest canopies (Ge et al., 2015). It is thus reasonable to speculate that the genetic differentiation pattern of *A. argotaenia* is highly linked to restricted between-population gene flow (genetic exchange *via* pollen and seed). Moreover, the establishment of climate- or habitat-linked genotypes should also be considered, since we used functional markers to perform studies (Jump and Peñuelas, 2005; Ortego et al., 2012).

## Conclusion

We generated the leaf transcriptome of *A. argotaenia* by using Illumina sequencing technology. A total of 62,896 unigenes were assembled, annotated, and classified. Based on the transcriptome data, 5510 EST-SSRs were identified from 4830 SSR-containing unigene sequences. Among them, 60 were randomly selected for the development of potential functional markers. Consequently, 22 polymorphic EST-SSR markers were developed and used to reveal a moderate level of functional diversity, along with marked genetic structure and the lack of genetic bottleneck, in *A. argotaenia*. This study has provided effective EST-SSR markers for measuring the evolutionary potential of *A. argotaenia* in response to environmental changes.

## Data Availability Statement

All Illumina clean data generated for this study was deposited at the Sequence Read Archive (SRA) of the National Center for Biotechnology Information (https://www.ncbi.nlm.nih.gov/sra/SRX3296043[accn]). The Bioproject number and Biosample number for clean data are PRJNA413732 and SAMN07764634, respectively. Thirty-eight EST-SSR sequences generated for this study were deposited at GenBank with accession numbers MG209531-MG209568.

## Author Contributions

The author XR conducted the experiments and ZW completed the data analysis. The two authors contributed equally to this work. YS designed the experiments and wrote the manuscript, and TW corrected the manuscript.

## Funding

This work was supported by the National Natural Science Foundation of China (31370364, 31570652, 31670200, 31770587, and 31872670); the Natural Science Foundation of Guangdong Province, China (2016A030313320 and 2017A030313122); Science and Technology Planning Project of Guangdong Province, China (2017A030303007); Project of Department of Science and Technology of Shenzhen City, Guangdong, China (JCYJ20160425165447211, JCYJ20170413155402977, and JCYJ20170818155249053); and Science and Technology Planning Project of Guangzhou City, China (201804010389).

## Conflict of Interest

The authors declare that the research was conducted in the absence of any commercial or financial relationships that could be construed as a potential conflict of interest.

## Supplementary Material

Supplementary Figure 1 Length distribution of assembled unigenes generated from *A. argotaenia* transcriptome.

Supplementary Figure 2 Functional classification of *A. argotaenia* unigenes based on KOG annotation.

Supplementary Figure 3 Functional classification of *A. argotaenia* unigenes based on KEGG annotation.

Supplementary Figure 4 The optimum number of clusters (*K*) estimated with STRUCTURE analysis based on Ln*P*(*D*) (A) and hoc statistic Δ*K* (B).

Supplementary Figure 5 Amplification products generated using EST-SSR primer pair, SHS-26622, were separated by electrophoresis in 6% denaturing polyacrylamide gel. The expected allele size was 228 bp and the annealing temperature was 57°C. Lanes 1-15, 16-28, 29-40, and 41-56 were products of *A. argotaenia* individuals from populations JQS (Jiuqushui), CP (Chuanping), QNS (Qiniangshan), and WGS (Wugongshan), respectively; Lane M: 50 bp ladder.
