# Supplementary figures and images for: Characterization and Application of EST-SSR Markers Developed From the Transcriptome of Amentotaxus argotaenia (Taxaceae), a Relict Vulnerable Conifer
Source: Front Genet. 2019 Oct 18;10:1014. doi: 10.3389/fgene.2019.01014 (PMC6813739; doi:10.3389/fgene.2019.01014)

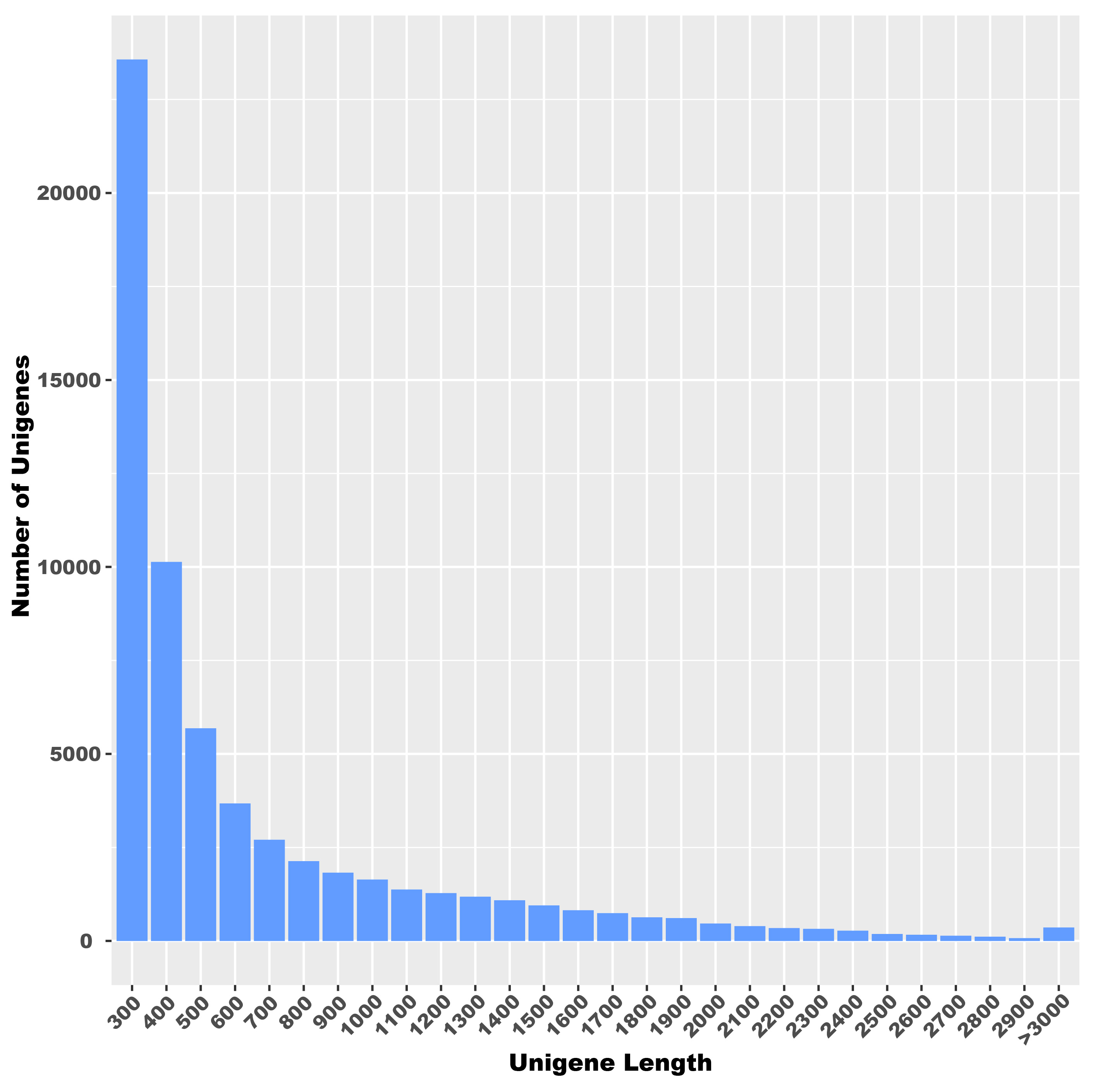

Supplement: Supplementary Figure 1 — Length distribution of assembled unigenes generated from A. argotaenia transcriptome. [file Image_1.jpeg]

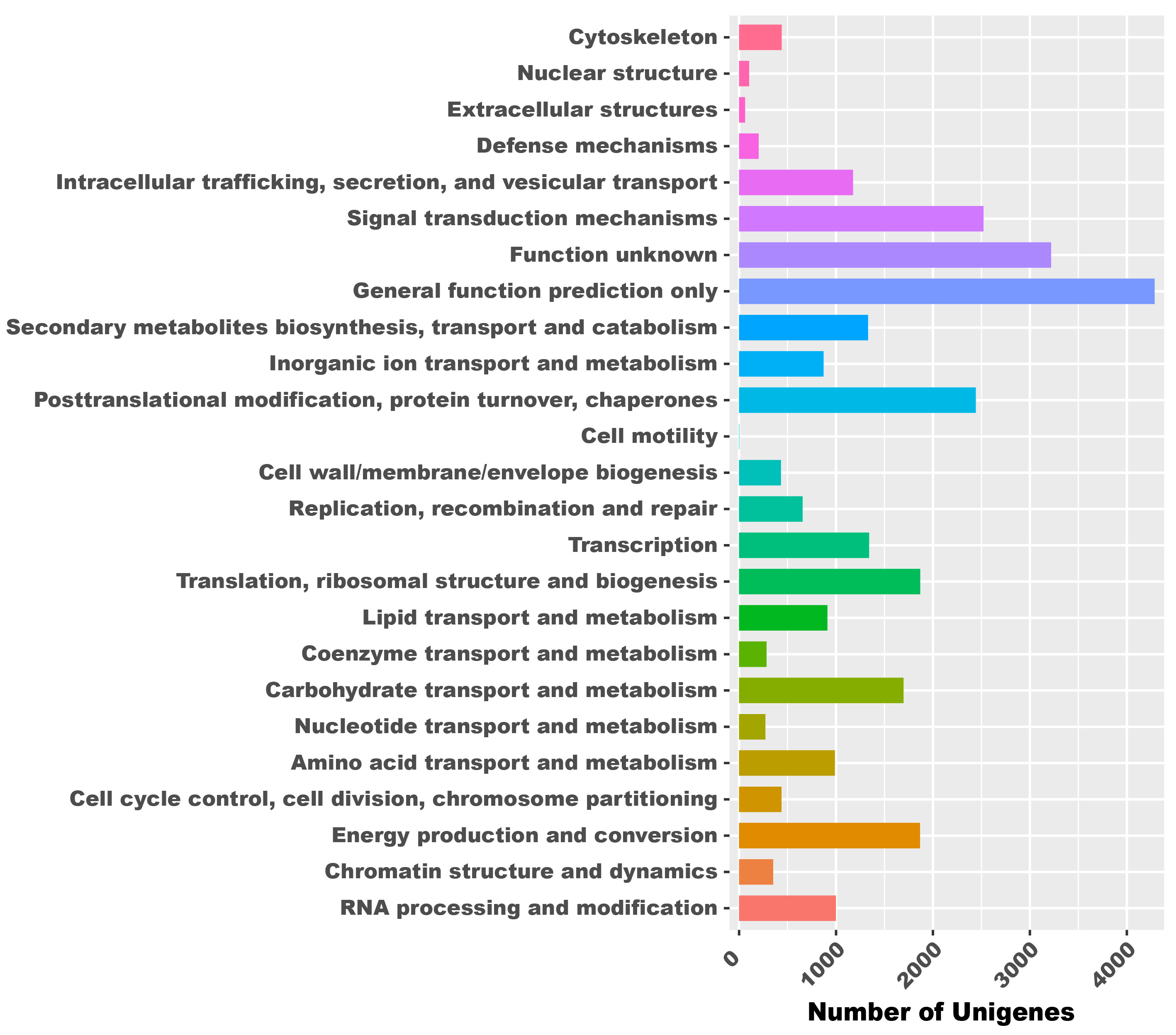

Supplement: Supplementary Figure 2 — Functional classification of A. argotaenia unigenes based on KOG annotation. [file Image_2.jpeg]

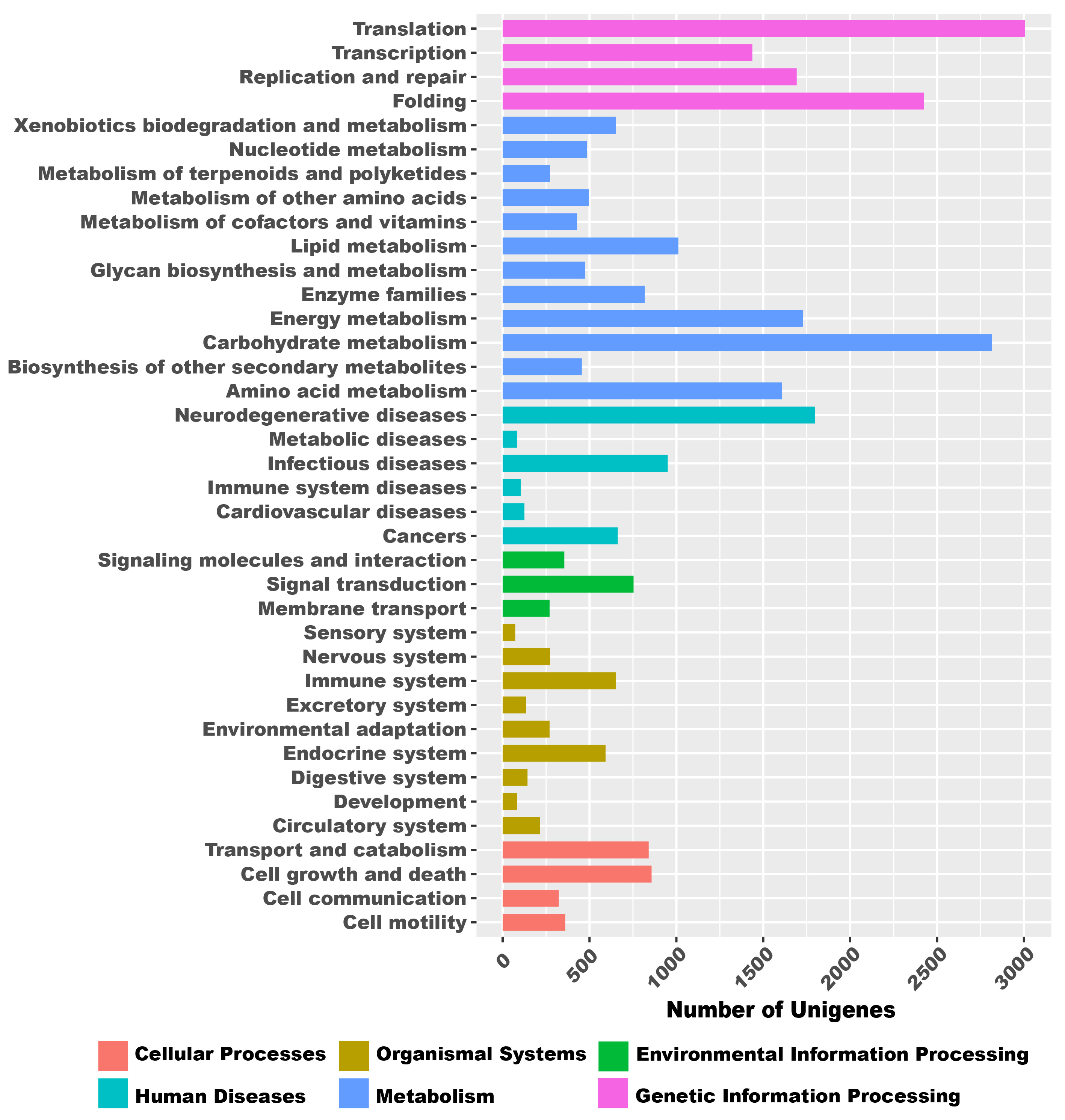

Supplement: Supplementary Figure 3 — Functional classification of A. argotaenia unigenes based on KEGG annotation. [file Image_3.jpeg]

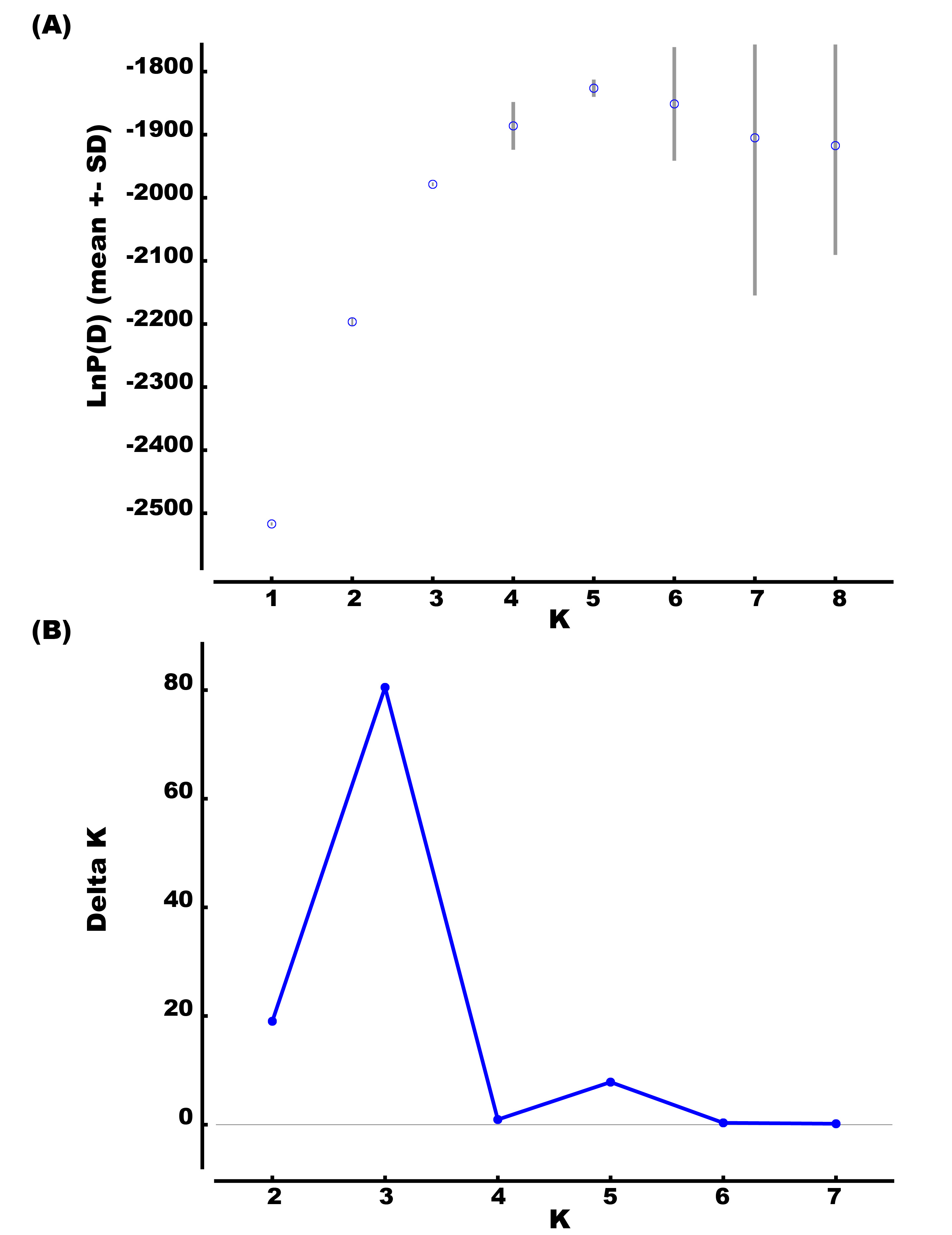

Supplement: Supplementary Figure 4 — The optimum number of clusters (K) estimated with STRUCTURE analysis based on LnP(D) (A) and hoc statistic ΔK (B). [file Image_4.jpeg]

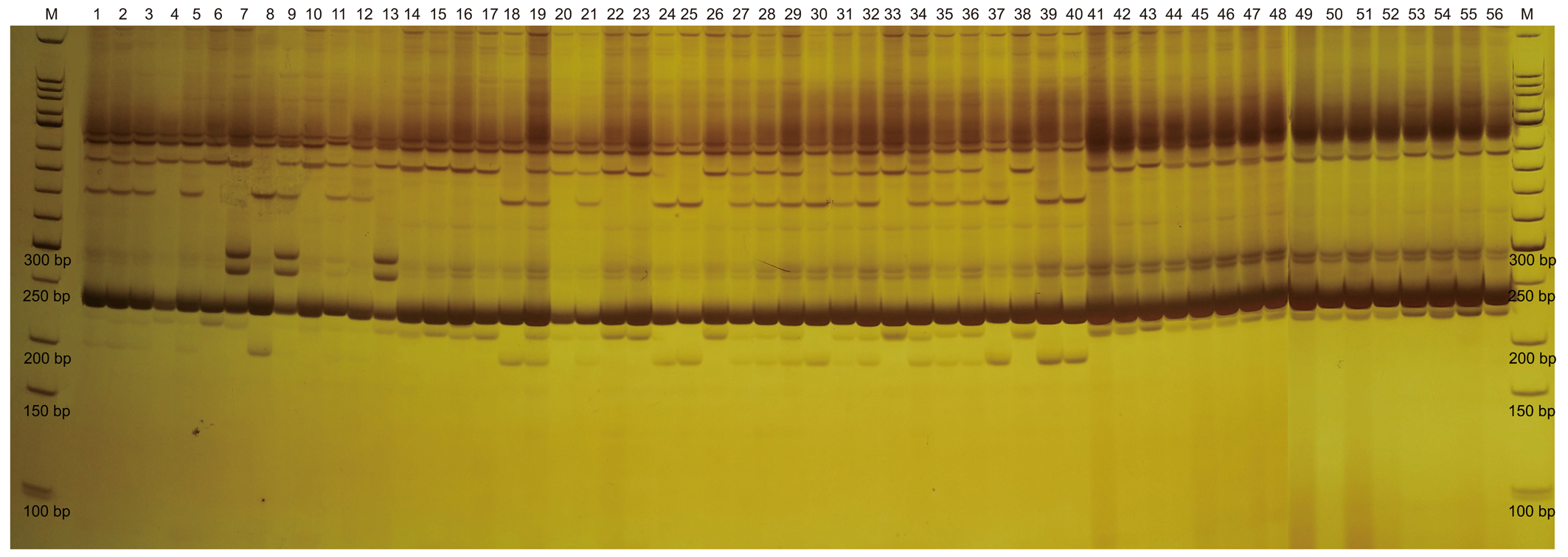

Supplement: Supplementary Figure 5 — Amplification products generated using EST-SSR primer pair, SHS-26622, were separated by electrophoresis in 6% denaturing polyacrylamide gel. The expected allele size was 228 bp and the annealing temperature was 57°C. Lanes 1-15, 16-28, 29-40, and 41-56 were products of A. argotaenia individuals from populations JQS (Jiuqushui), CP (Chuanping), QNS (Qiniangshan), and WGS (Wugongshan), respectively; Lane M: 50 bp ladder. [file Image_5.jpeg]
