# Supplementary material for: Characterization and Application of EST-SSR Markers Developed From the Transcriptome of Amentotaxus argotaenia (Taxaceae), a Relict Vulnerable Conifer
Source: Front Genet. 2019 Oct 18;10:1014. doi: 10.3389/fgene.2019.01014 (PMC6813739; doi:10.3389/fgene.2019.01014)
Supplement: Supplementary file 6 [file Table_1.docx]

Table S1 Voucher and location information for species and populations used in this study.^a^

| Species | *N* | Population | Geographic coordinates | Altitude (m) | Voucher no. |
| --- | --- | --- | --- | --- | --- |
| *Amentotaxus argotaenia* | 15 | Jiuqushui, Hunan (JQS) | 26°34ʹ02.10ʹʹ N, 114°04ʹ42.27ʹʹ E | 845 | LXP134282 |
| *A. argotaenia* | 13 | Chuanping, Jiangxi (CP) | 26°45ʹ26.36ʹʹ N, 114°10ʹ12.63ʹʹ E | 608 | LXP1307901 |
| *A. argotaenia* | 16 | Wugongshan, Jiangxi (WGS) | 27°27ʹ53.10ʹʹ N, 114°09ʹ56.63ʹʹ E | 1000 | WGS1327 |
| *A. argotaenia* | 12 | Qiniangshan, Guangdong (QNS) | 22°31ʹ31.52ʹʹ N, 114°32ʹ27.37ʹʹ E | 747 | SZ12264 |

*N*, number of individuals; ^a^, All specimens are deposited at the herbarium of Sun Yat-sen University (SYS), Guangzhou, China.
