# Supplementary material for: Characterization and Application of EST-SSR Markers Developed From the Transcriptome of Amentotaxus argotaenia (Taxaceae), a Relict Vulnerable Conifer
Source: Front Genet. 2019 Oct 18;10:1014. doi: 10.3389/fgene.2019.01014 (PMC6813739; doi:10.3389/fgene.2019.01014)
Supplement: Supplementary file 7 [file Table_2.docx]

Table S2 Results of molecular variance analysis for *A. argotaenia* populations.

| Source of variation | d.f. | Sum of squares | Variance components | Percentage of variation | F statistics |
| --- | --- | --- | --- | --- | --- |
| Among groups | 2 | 122.769 | 1.11386 | 17.83 | *F*ct =0.17828 |
| Among populations within groups | 1 | 22.536 | 0.64793 | 10.37 | *F*sc =0.12620 |
| Within populations | 108 | 484.508 | 4.48618 | 71.80 | *F*st =0.28198 |
| Total | 111 | 629.812 | 6.24797 |  |  |
