# Supplementary material for: Characterization and Application of EST-SSR Markers Developed From the Transcriptome of Amentotaxus argotaenia (Taxaceae), a Relict Vulnerable Conifer
Source: Front Genet. 2019 Oct 18;10:1014. doi: 10.3389/fgene.2019.01014 (PMC6813739; doi:10.3389/fgene.2019.01014)
Supplement: Supplementary file 8 [file Table_3.docx]

Table S3 Bottleneck analysis for four *A. argotaenia*. populations

| Population | Wilcoxon’s signed rank test | | | |
| --- | --- | --- | --- | --- |
|  | *P* value (one tail for heterozygosity deficiency) | *P* value (one tail for heterozygosity excess) | *P* value (two tails for heterozygosity deficiency or heterozygosity excess) | Mode-shift test |
| JQS | 0.83764 | 0.17327 | 0.34655 | L-shape |
| CP | 0.72706 | 0.28530 | 0.57060 | shifted mode |
| QNS | 0.81314 | 0.19629 | 0.39258 | L-shape |
| WGS | 0.94447 | 0.05967 | 0.11934 | L-shape |
| Total | 0.07799 | 0.92674 | 0.15597 | L-shape |

*P* values are determined by Wilcoxon’s signed rank test under the two-phase mutation model (TPM).
