# Supplementary material for: Characterization and Application of EST-SSR Markers Developed From the Transcriptome of Amentotaxus argotaenia (Taxaceae), a Relict Vulnerable Conifer
Source: Front Genet. 2019 Oct 18;10:1014. doi: 10.3389/fgene.2019.01014 (PMC6813739; doi:10.3389/fgene.2019.01014)
Supplement: Supplementary file 9 [file Table_4.docx]

Table S4 Comparison of genetic diversity and population differentiation in different gymnosperm species.

| Species | Family | *N*_a_ | *H*_e_ | *H*_o_ | PIC | *PPL* | *F*st | Reference |
| --- | --- | --- | --- | --- | --- | --- | --- | --- |
| EST-SSRs |  |  |  |  |  |  |  |  |
| *Pinus bungeana* | Pinaceae |  | 0.1994 | 0.2214 |  | 8.14% | 0.2152 | *Duan et al., 2017* |
| *Pinus dabeshanensis* | Pinaceae | 3.23 | 0.458 | 0.481 |  |  | 0.028 | *Xiang et al., 2015* |
| *Amentotaxus argotaenia* | Taxaceae | 2.348 | 0.394 | 0.297 | 0.328 | 100% |  | *Li et al., 2016* |
| *Amentotaxus formosana* | Taxaceae | 1.783 | 0.1993 | 0.053 | 0.1693 | 60.87% |  | *Li et al., 2016* |
| *Amentotaxus yunnanensis* | Taxaceae | 2.478 | 0.3343 | 0.2683 | 0.291 | 73.91% |  | *Li et al., 2016* |
| *Amentotaxus poilanei* | Taxaceae | 2.174 | 0.347 | 0.359 | 0.291 | 82.61% |  | *Li et al., 2016* |
| *Amentotaxus argotaenia* | Taxaceae | 2.682 | 0.39 | 0.25 | 0.455 | 57.89% | 0.28198 | This study |
| SSRs |  |  |  |  |  |  |  |  |
| *Abies ziyuanensis* | Pinaceae |  | 0.337 | 0.319 |  | 62.50% | 0.25 | *Tang et al., 2008* |
| *Pinus krempfii* | Pinaceae |  | 0.229 | 0.303 |  | 51.47% | 0.119 | *Phong et al., 2015* |
| *Pinus strobus* | Pinaceae |  | 0.802 | 0.704 |  |  | 0.084 | *Mehes, Nkongolo & Michael, 2009* |
| *Pinus monticola* | Pinaceae |  | 0.808 | 0.683 |  |  | 0.057 | *Mehes, Nkongolo & Michael, 2009* |
| *Pinus sylvestris* | Pinaceae |  | 0.787 | 0.947 |  |  | 0.007 | *Pazouki et al., 2016* |
| *Podocarpus sellowii* | Podocarpaceae |  | 0.302 | 0.393 |  | 42.86% | 0.259 | *Dantas et al., 2015* |
| *Cephalotaxus oliveri* | Cephalotaxaceae |  | 0.413 | 0.502 |  | 68.42% | 0.639 | *Pan et al., 2011* |
| *Amentotaxus argotaenia* | Taxaceae |  | 0.61 | 0.12 |  |  | 0.22 | *Ge et al., 2015* |
| *Amentotaxus yunnanensis* | Taxaceae |  | 0.36 | 0.23 |  |  | 0.20 | *Ge et al., 2015* |
| *Amentotaxus argotaenia* | Taxaceae | 5.304 | 0.66 | 0.859 | 0.707 | 82.14% | 0.1304 | *Huang et al., 2018* |
| *Amentotaxus yunnanensis* | Taxaceae | 6.389 | 0.701 | 0.915 | 0.651 | 100% |  | *Huang et al., 2018* |
| *Taxus yunnanensis* | Taxaceae |  | 0.37 | 0.107 |  |  | 0.196 | *Miao et al., 2014* |
| *Taxus wallichiana var. mairei* | Taxaceae |  | 0.538 | 0.39 |  |  | 0.159 | *Zhang & Zhou, 2013* |

*N*_a_, number of alleles per locus; *H*_e_, expected heterozygosity; *H*_o_, observed heterozygosity; PIC, polymorphism information content; *PPL*, percentage of polymorphic loci; *F*st, genetic differentiation.

**Reference**

Dantas LG, Esposito T, de Sousa AC, Félix L, Amorim LL, Benko-lseppon AM, Batalha-Filho H, Pedrosa-Harand A. 2015. Low genetic diversity and high differentiation among relict populations of the neotropical gymnosperm *Podocarpus sellowii* (Klotz.) in the Atlantic Forest. *Genetica* 143:21-30 DOI: 10.1007/s10709-014-9809-y.

Duan D, Jia Y, Yang J, Li ZH. 2017. Genetic diversity analysis of *Pinus bungeana* natural populations with EST-SSR markers. *Genes* 8:393 DOI: 10.3390/genes8120393.

Ge XJ, Hung KH, Ko YZ, Hsu TW, Gong X, Chiang TY, Chiang YC. 2015. Genetic Divergence and Biogeographical Patterns in *Amentotaxus argotaenia* Species Complex. *Plant Molecular Biology Reporter* 33:264-280 DOI: 10.1007/s11105-014-0742-0.

Huang QQ, Wang Z, Wang T, Su YJ. 2018. Development and characterization of 23 polymorphic microsatellite loci for *Amentotaxus argotaenia* (Taxaceae), a relict vulnerable species. *Applications in Plant Sciences* 6:e01149 DOI: 10.1002/aps3.1149.

Li CY, Chiang TY, Chiang YC, Hsu HM, Ge XJ, Huang CC, Chen CT, Hung KH. 2016. Cross-species, amplifiable EST-SSR markers for *Amentotaxus* species obtained by next-generation sequencing. *Molecules* 21:67 DOI: 10.3390/molecules21010067.

Mehes M, Nkongolo KK, Michael P. 2009. Assessing genetic diversity and structure of fragmented populations of eastern white pine (*Pinus strobus*) and western white pine (*P. monticola*) for conservation management. *Journal of Plant Ecology* 2:143-151 DOI: 10.1093/jpe/rtp016.

Miao YC, Lang XD, Zhang ZZ, Su JR. 2014. Phylogeography and genetic effects of habitat fragmentation on endangered *Taxus yunnanensis* in southwest China as revealed by microsatellite data. *Plant Biology* 16:365-374 DOI: 10.1111/plb.12059.

Pan HW, Guo YR, Su YJ, Wang T. 2011. Development of microsatellite loci for *Cephalotaxus oliveri* (Cephalotaxaceae) and cross-amplification in *Cephalotaxus*. *American Journal of Botany* 98:e229-32 DOI: 10.3732/ajb.1100128.

Pazouki L, Shanjani PS, Fields PD, Martins K, Suhhorutšenko M, Viinalass H. 2016. Large within-population genetic diversity of the widespread conifer *Pinus sylvestris*, at its soil fertility limit characterized by nuclear and chloroplast microsatellite markers. *European Journal of Forest Research* 135:161-177 DOI: 10.1007/s10342-015-0928-5.

Phong DT, Lieu TT, Hien VT, Hiep NT. 2015. Genetic diversity of the endemic flat-needle pine *Pinus krempfii* (Pinaceae) from Vietnam revealed by SSR markers. *Genetics & Molecular Research Gmr* 14:7727-7739. DOI: 10.4238/2015.July.13.19.

Tang S, Dai W, Li M, Zhang Y, Geng Y, Wang L, Zhong Y. 2008. Genetic diversity of relictual and endangered plant *Abies ziyuanensis* (Pinaceae) revealed by AFLP and SSR markers. *Genetica* 133:21-30 DOI: 10.1007/s10709-007-9178-x.

Xiang XY, Zhang ZX, Wang ZG, Zhang XP, Wu GL. 2015. Transcriptome sequencing and development of EST-SSR markers in *Pinus dabeshanensis*, an endangered conifer endemic to China. *Molecular Breeding* 35:158 DOI: 10.1007/s11032-015-0351-0.

Zhang DQ, Zhou N. 2013. Genetic diversity and population structure of the endangered conifer *Taxus wallichiana* var. *mairei* (Taxaceae) revealed by Simple Sequence Repeat (SSR) markers. Biochemical Systematics and Ecology 49:107-114 DOI: 10.1016/j.bse.2013.03.030.
